# Discovery of viruses and bacteria associated with swine respiratory disease on farms at a nationwide scale in China using metatranscriptomic and metagenomic sequencing

**DOI:** 10.1128/msystems.00025-25

**Published:** 2025-01-30

**Authors:** Xi Huang, Xinzhi Yao, Wenbo Song, Mengfei Zhao, Zhanwei Zhu, Hanyuan Liu, Xiaorong Song, Jingwen Huang, Yongrun Chen, Zihao Wang, Changjiang Peng, Wenqing Wu, Hao Yang, Lin Hua, Huanchun Chen, Bin Wu, Zhong Peng

**Affiliations:** 1 National Key Laboratory of Agricultural Microbiology, College of Veterinary Medicine, Huazhong Agricultural University, Wuhan, China; 2 Hubei Hongshan Laboratory, Wuhan, China; 3 Frontiers Science Center for Animal Breeding and Sustainable Production, The Cooperative Innovation Center for Sustainable Pig Production, Wuhan, China; 4 College of Informatics, Hubei Key Laboratory of Agricultural Bioinformatics, Huazhong Agricultural University, Wuhan, China; University of California San Diego, La Jolla, California, USA

**Keywords:** swine respiratory disease, nationwide genomic surveillance, virome, bacteriome, resistome, metatranscriptomic sequencing, metagenomic sequencing, China

## Abstract

Respiratory disease (RD) is a worldwide leading threat to the pig industry, but there is still limited understanding of the pathogens associated with swine RD. In this study, we conducted a nationwide genomic surveillance on identifying viruses, bacteria, and antimicrobial resistance genes (ARGs) from the lungs of pigs with RD in China. By performing metatranscriptomic sequencing combined with metagenomic sequencing, we identified 21 viral species belonging to 12 viral families. Among them, porcine reproductive and respiratory syndrome virus, influenza A virus, herpes virus, adenovirus, and parvovirus were commonly identified. However, emerging viruses, such as Getah virus and porcine respiratory coronaviruses, were also characterized. Apart from viruses, a total of 164 bacterial species were identified, with *Streptococcus* suis, *Mycoplasma hyorhinis*, *Mycoplasma hyopneumoniae*, *Glaesserella parasuis*, and *Pasteurella multocida* being frequently detected in high abundances. Notably, *Escherichia coli*, *Enterococcus faecalis*, *Staphylococcus aureus*, and *Klebsiella pneumoniae* were also highly detected. Our further analysis revealed a complex interaction between the identified pathogens in swine RD. We also conducted retrospectively analyses to demonstrate the prevalent viral genotypes or bacterial serotypes associated with swine RD in China. Finally, we identified 48 ARGs, which conferred resistance to 13 predicted antimicrobial classes, and many of these ARGs were significantly associated with a substantial number of mobile genetic elements, including transposons (e.g., *tnp*AIS1, *tnp*A1353, *int*3, and *IS*Cau1) and plasmids (e.g., Col(BS512), Col(YC)]. These findings will contribute to further understanding the etiology, epidemiology, and microbial interactions in swine RD, and may also shed a light on the development of effective vaccines.

**IMPORTANCE:**

In this study, we identified viruses and bacteria from the lungs of pigs with RD in China at a nationwide farm scale by performing metatranscriptomic sequencing combined with metagenomic sequencing. We also demonstrated the complex interactions between different viral and/or bacterial species in swine RD. Our work provides a comprehensive knowledge about the etiology, epidemiology, and microbial interactions in swine RD and data reference for the research and development of effective vaccines against the disease.

## INTRODUCTION

Domestic pigs (*Sus scrofa domesticus*) are among the most commonly raised animals worldwide for food production. In 2023, the global pig population was estimated to be over 778 million, producing more than 120 millionmetric tons of pork (1, 2). However, the pig industry worldwide faces significant challenges due to infectious diseases. Respiratory diseases, caused by both viral and bacterial pathogens, represent significant health concerns in pigs raised in intensive farming systems (3). Recurrent outbreaks of viral pathogens, such as porcine reproductive and respiratory syndrome virus (PRRSV), porcine circovirus (PCV), and swine influenza virus (SIV), have led to considerable mortality, trade restrictions, and a marked decline in productivity (4–6). Bacterial pathogens, such as *Actinobacillus pleuropneumonia*, *Streptococcus suis,* and *Mycoplasma hyopneumoniae*, also play a critical role, often acting as primary etiological agents or contributing to secondary infections following viral exposure, exacerbating disease severity (7, 8). These pathogens exhibit considerable genetic diversity and frequent mutations, which not only heighten the risk of transmission but also complicate diagnostic and control strategies. Additionally, many pathogens prevalent in pig farms are zoonotic, meaning they can be transmitted from animals to humans (9). The presence of these zoonotic pathogens not only poses a risk to pig health but also threatens food safety and human health.

On farms, respiratory infections in pigs frequently occur as co-infections led by multiple pathogens. For example, viral agents, such as PRRSV and PCV2, along with bacterial agents, like *Streptococcus suis* and *Glaesserella parasuis*, are commonly identified together in pigs exhibiting respiratory symptoms (10, 11). The co-infection of these pathogens can result in complex synergistic interactions, prolonging the duration of the disease and exacerbating the severity of clinical symptoms. China is the largest pig-rearing and pork-consuming country in the world, accounting for more than 50% of the worldwide pork production and consumption (1, 2). Despite significant achievements made by the Chinese pig industry over the past 70 years, infectious diseases continue to pose the most significant threat to its development (12). Respiratory disease, in particular, have resulted in significant morbidity, mortality, and economic losses (4). However, there is still limited understanding of the pathogens associated with respiratory disease on Chinese pig farms, including their prevalent serotypes or genotypes, and co-infection. Recently, high-throughput sequencing has emerged as a powerful tool for large-scale identification of pathogens and antimicrobial resistance genes (ARGs) (13–15). In this study, we conducted a comprehensive genomic surveillance of pathogens in the lungs of pigs that succumbed to respiratory disease across China using high-throughput sequencing. Additionally, we investigated the antibiotic resistome in the lower respiratory tract of pigs affected by respiratory disease. Our objective was to provide comprehensive knowledge regarding the pathogens associated with respiratory disease and their interactions on pig farms.

## RESULTS

### Identification of pathogens associated with pig respiratory disease

**Fig 1:**
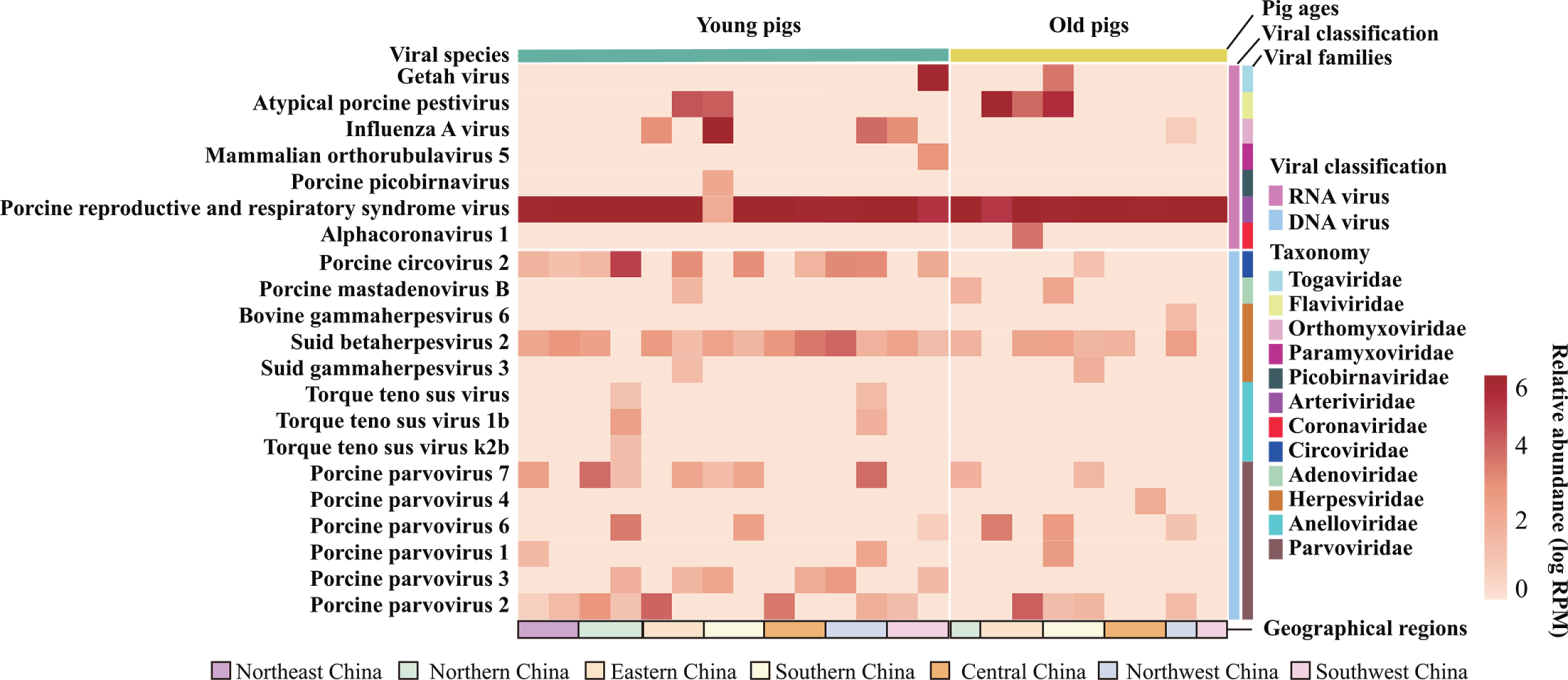
Viral species identified by metatranscriptomic sequencing and metagenomic sequencing from the lung tissues of pigs with respiratory disease in China. A heatmap showing the relative abundances of different viral species in the lungs of pigs collected from different regions in China, with relative abundance values expressed as log reads per million (RPM).

Metatranscriptomic and metagenomic sequencing determined a massive sequencing data from the sample pools (Fig. 1). These data were then subjected to virome, bacteriome, and resistome analyses. Despite a mass of viral and bacterial species identified, we only considered those that had been reported to be associated with mammal respiratory disease. Through this approach, a total of 21 viral species belonging to 12 viral families were characterized (Fig. 1; Table S1). These 21 viral species consisted of 7 RNA viruses and 14 DNA viruses.

**Fig 2:**
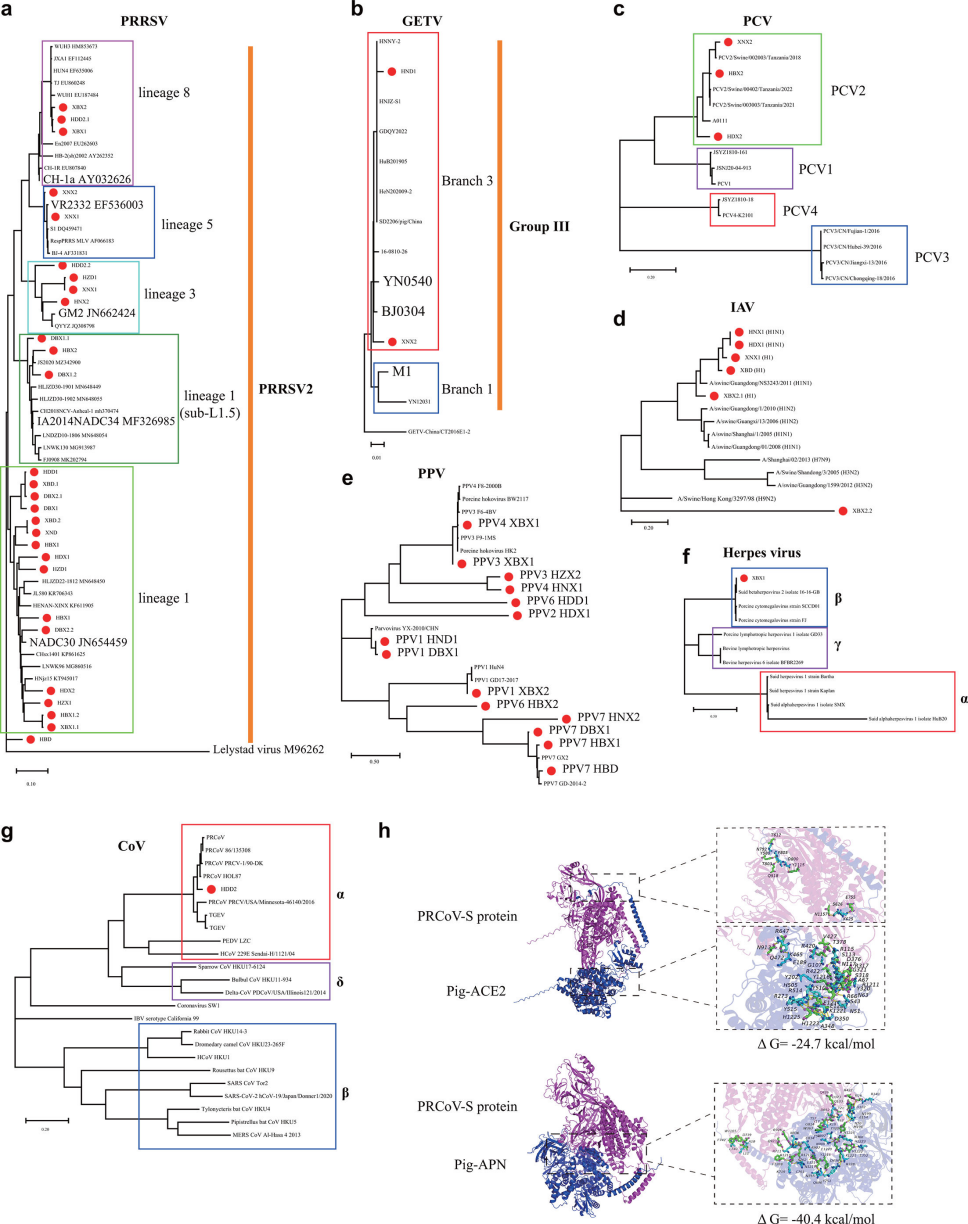
Phylogenetic analysis of different viral species identified in this study. (**a**) Phylogenetic relationships of porcine reproductive and respiratory syndrome virus (PRRSV) strains. PRRSV strains identified in this study were indicated using small red circles. The tree was generated based on the complete genome sequences. PRRSV1 referred to PRRSV genotype I, while PRRSV2 referred to PRRSV genotype II. (**b**) Phylogenetic relationships of Getah virus (GETV) strains. GETV strains identified in this study were indicated using small red circles. The tree was generated based on the complete genome sequences. (**c**) Phylogenetic relationships of porcine circovirus (PCV) strains. PCV strains identified in this study were indicated using small red circles. The tree was generated based on the complete genome sequences. (**d**) Phylogenetic relationships of influenza A virus (IAV) strains. IAV strains identified in this study were indicated using small red circles. The tree was generated based on the nucleotide sequences of hemagglutinin (HA) gene. (**e**) Phylogenetic relationships of porcine parvovirus (PPV) strains. PPV strains identified in this study were indicated using small red circles. The tree was generated based on the complete genome sequences. (**f**) Phylogenetic relationships of porcine herpes virus strains. Herpes virus strains identified in this study were indicated using small red circles. The tree was generated based on the complete genome sequences. (**g**) Phylogenetic relationships of coronavirus (CoV) strains. Porcine respiratory CoV (PRCoV) strain identified in this study was indicated using small red circles. The tree was generated based on the nucleotide sequences of spike (S) gene. (**h**) Molecular docking of the spike protein of PRCoV strain HDD2 with porcine ACE2 protein and porcine APN protein.

PRRSV was found to be the virus with broad-range regions and high abundance of detection in the lung tissues of both young and old pigs (Fig. 1). Our further analysis revealed that the PRRSV strains identified in the lungs of pigs were PRRSV2, and they distributed in four different lineages (L1, L3, L5, and L8) (Fig. 2a). However, the majority of them were lineage 1 strains, represented by the emerging PRRSV strains NADC-30 and NADC-34 (Fig. 2a). In addition, Getah virus (GETV) was also detected from the lungs of pigs from south China, and they belonged to group III (Fig. 1 and 2b). PCV2 was the only characterized PCV in the lung tissues (Fig. 2c). Additionally, influenza A virus (IAV), herpes virus, adenovirus, and parvovirus were also common (Fig. 2d through f). Particularly, six types of parvoviruses (PPV1, PPV2, PPV3, PPV4, PPV6, and PPV7) were detected (Fig. 2e). We also identified a α coronavirus (strain HDD2) in the lungs from old pigs in eastern China, and this virus was closely related to the other porcine respiratory coronaviruses (PRCoV) characterized before (Fig. 2g). Molecular docking revealed the spike protein of HDD2 was able to dock with porcine ACE2 protein (Δ*G* = −24.7 kcal/mol) and porcine APN protein (Δ*G* = −40.4 kcal/mol) (Fig. 2h). The complete sequence of a Zhejiang porcine bastro-like virus was also generated from the lungs of old pigs in eastern China (Fig. S2).

**Fig 3:**
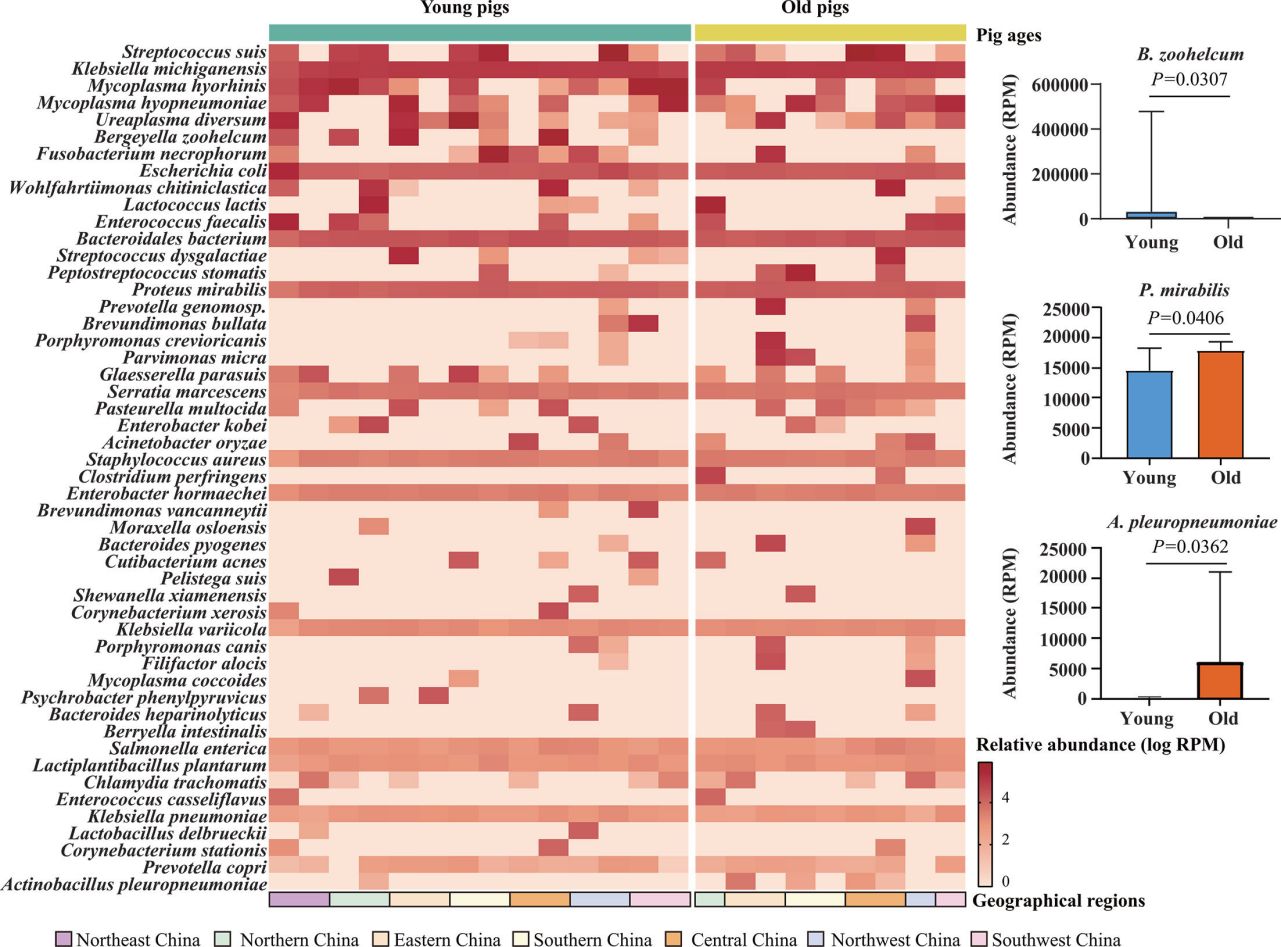
Bacterial species identified by metagenomic sequencing and metagenomic sequencing from the lung tissues of pigs with respiratory disease in China. The relative abundance of different bacterial species in the lungs of pigs was displayed using a heatmap, with relative abundance values expressed as log RPM. Abundances of *Bergeyella zoohelcum, Proteus mirabilis*, and *Actinobacillus pleuropneumoniae* in the lungs of old and young pigs were displayed using column charts. *P*-values were determined using the Mann–Whitney U test for multiple comparisons between groups. Data represent mean ± SD.

A total of 164 bacterial species were identified, and *S. suis* displayed the highest abundance of detection (Fig. 3; Table S2). Other known bacterial species frequently associated with swine respiratory disease, including *Mycoplasma hyorhinis*, *M. hyopneumoniae*, *G. parasuis*, *Pasteurella multocida*, and *A. pleuropneumoniae*, were also detected in high abundance (Fig. 3). Additionally, *Escherichia coli*, *Enterococcus faecalis*, *Staphylococcus aureus*, and *Klebsiella pneumoniae* showed high detection levels (Fig. 3). A significantly higher abundance of *Bergeyella zoohelcum* was observed in the lungs of young pigs compared with older pigs (*P* = 0.0307). However, the abundance of *Proteus mirabilis* (*P* = 0.0406) and *A. pleuropneumoniae* (*P* = 0.0362) was higher in the lungs of older pigs (Fig. 3).

### Interactions between different pathogens

**Fig 4:**
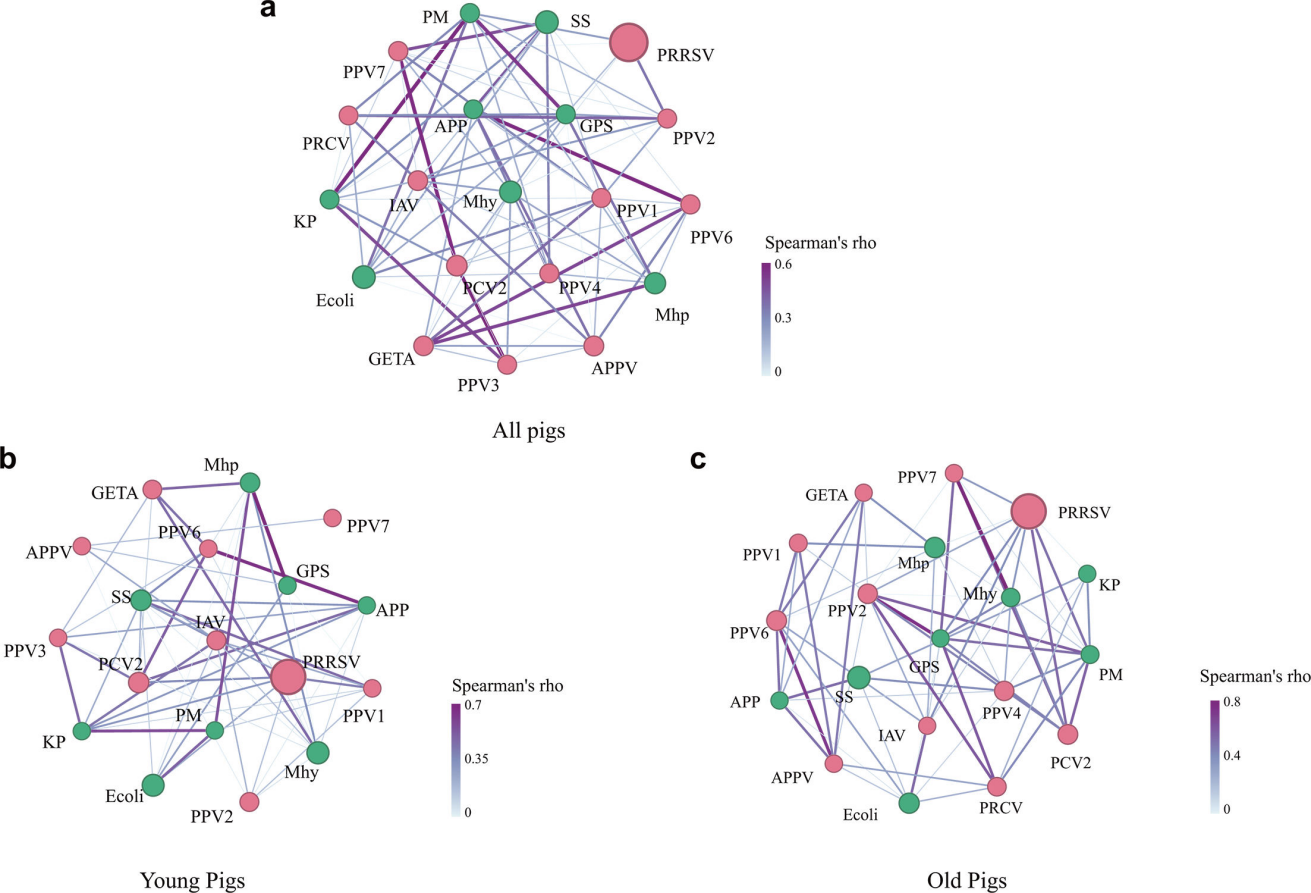
Correlations between different pathogenic microbes identified in the lungs of pigs with respiratory disease by metatranscriptomic sequencing and metagenomic sequencing. (**a**) Correlations between different pathogenic microbes identified in the lungs of all pigs with respiratory disease. (**b**) Correlations between different pathogenic microbes identified in the lungs of young pigs with respiratory disease. (**c**) Correlations between different pathogenic microbes identified in the lungs of old pigs with respiratory disease. The correlation network was generated based on Spearman’s correlation analysis. Viruses were depicted as pink nodes, while bacteria were represented as green nodes. The size of each node corresponds to the relative abundance of the respective pathogen, and the thickness and color intensity of each edge are proportional to the Spearman’s rho value. APP, *Actinobacillus pleuropneumoniae*; APPV, atypical porcine pestivirus; Ecoli, *Escherichia coli*; GETA, Getah virus; GPS, *Glaesserella parasuis*; IAV, influenza A virus; KP, *Klebsiella pneumoniae*; Mhp, *Mycoplasma hyopneumoniae*; Mhy, *Mycoplasma hyorhinis*; PCV2, porcine circovirus 2; PM, *Pasteurella multocida*; PPV1, porcine parvovirus 1; PPV2, porcine parvovirus 2; PPV3, porcine parvovirus 2; PPV4, porcine parvovirus 4; PPV6, porcine parvovirus 6; PPV7, porcine parvovirus 7; PRCV, porcine respiratory coronavirus; PRRSV, porcine reproductive and respiratory syndrome virus; SS, *Streptococcus suis*.

Coinfections between different pathogens are considered as the primary contributor to the development of porcine respiratory disease on farms (16). Therefore, we analyzed the interactions between the pathogens in the lungs of pigs that died from respiratory disease by using the Spearman correlation analysis, as previously described (17). This analysis demonstrated a complex interactive net between different pathogens (Fig. 4). Overall, PRRSV exhibited an interaction with a broad spectrum of microbes, including both viruses (e.g., GETV, PPV2) and bacteria (e.g., *S. suis*, *P. multocida*, *K. pneumoniae*, *M. hyorhinis*, *M. hyopneumoniae*, *G. parasuis*) (Fig. 4). Coinfections between GETV and *M. hyopneumoniae*, *G. parasuis*, *A. pleuropneumoniae*, PPV1, or PPV6 were also characterized (Fig. 4). Interactions between IAV and *A. pleuropneumoniae* or *G. parasuis* also existed in porcine respiratory disease (Fig. 4). Notably, a strong interaction between *P. multocida* and *E. coli* and/or *K. pneumoniae* was observed, even though reports on the role of *E. coli* and/or *K. pneumoniae* in porcine respiratory disease are still lacking (Fig. 4).

### Prevalence of viruses associated with pig respiratory disease on Chinese farms

**Fig 5:**
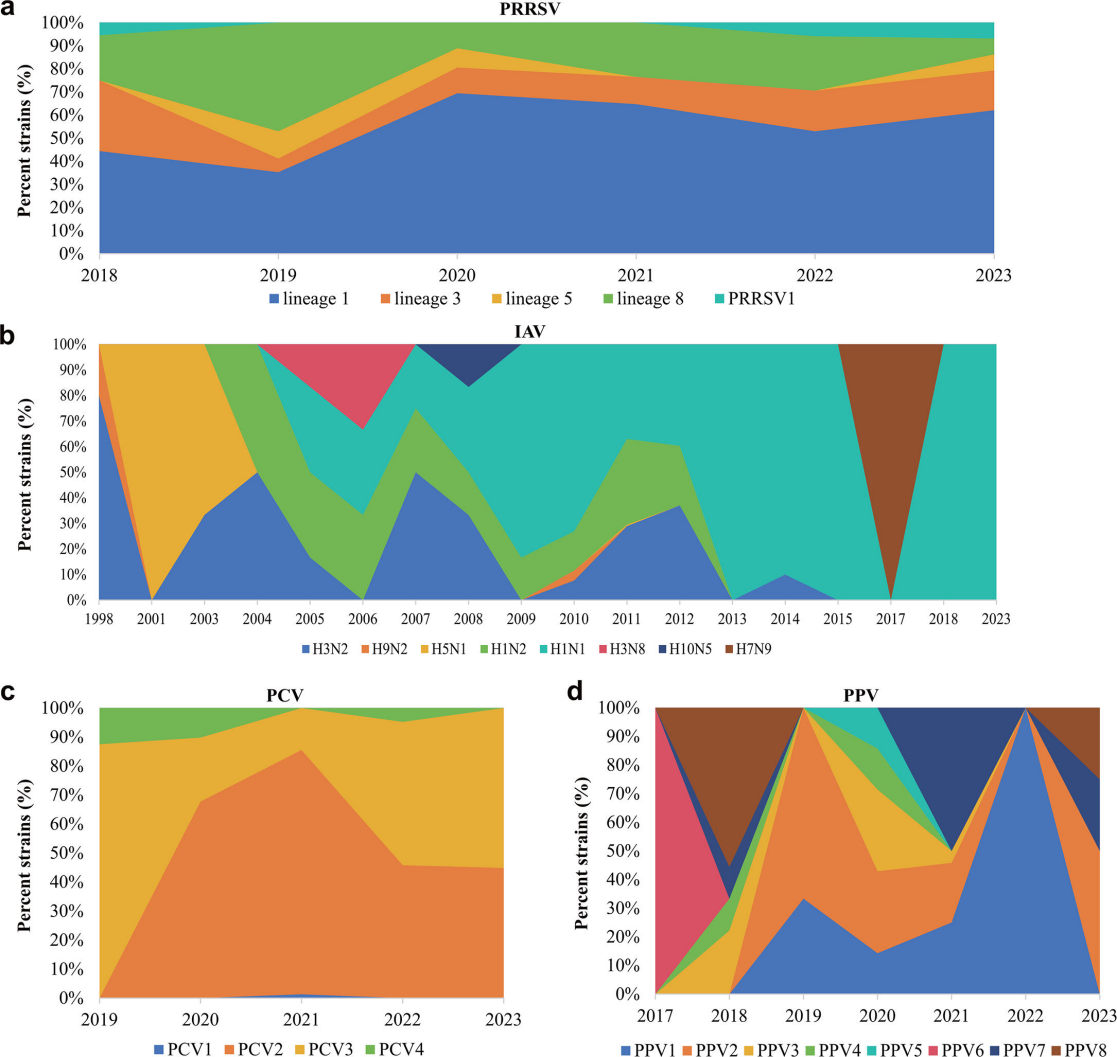
Change dynamics of different main viral species associated with swine respiratory disease in China as retrospectively determined based on published genome sequences in NCBI. (**a**) Change dynamics of porcine reproductive and respiratory syndrome virus (PRRS) on Chinese pig farms between 2018 and 2023 as retrospectively determined based on published genome sequences in NCBI. (**b**) Change dynamics of influenza A virus (IAV) on Chinese pig farms between 1998 and 2023 as retrospectively determined based on published genome sequences in NCBI. (**c**) Change dynamics of porcine circovirus (PCV) on Chinese pig farms between 2019 and 2023 as retrospectively determined based on published genome sequences in NCBI. (**d**) Change dynamics of porcine parvovirus (PPV) on Chinese pig farms between 2017 and 2023 as retrospectively determined based on published genome sequences in NCBI.

To understand the evolutionary and prevalent characteristics of main viral pathogens associated with porcine respiratory disease across China, published genome sequences of main viral pathogens identified in this study were downloaded from NCBI and set to conduct retrospectively phylogenetic analyses. Analysis of 170 published PRRSV complete genomes from China between 2018 and 2023 demonstrated that PRRSV2 (97.06%, 165/170) was still the predominant PRRSV type on pig farms in China, whereas PRRSV1 (2.94%, 5/170) was also existing (Fig. 5a; Table S3). Identified PRRSV2 strains included lineages L1, L3, L5, and L8, and L1 was predominantly prevalent in different years (Fig. 5a). The analysis of 424 genome sequences of IAV strains from pigs in China between 1998 and 2023 revealed a complex prevalence of this viral species (Fig. 5b; Table S4). Overall, H1N1 was the predominantly characterized serotype in China after 2009, but the other serotypes, such as H3N8, H10N5, and more recently, H7N9, were also identified (Fig. 5b). Analysis of 685 genome sequences of PCV strains between 2019 and 2023 showed that PCV2 was the predominant PCV type but the prevalence of PCV3 was increasing after 2022 (Fig. 5c; Table S5). Notably, PCV4 was also identified on Chinese pig farms. The analysis of 65 PPV genome sequences from strains of swine between 2017 and 2023 also indicated a complex prevalence of PPV on Chinese pig farms, and PPV1 was predominantly identified in recent years (Fig. 5d; Table S6). There were only 45 published genome sequences of GETV strains from pigs of China in NCBI, and the analysis of these 45 genome sequences showed all of them belonged to GIII (Table S7).

### Prevalence of bacteria associated with pig respiratory disease on Chinese farms

**Fig 6:**
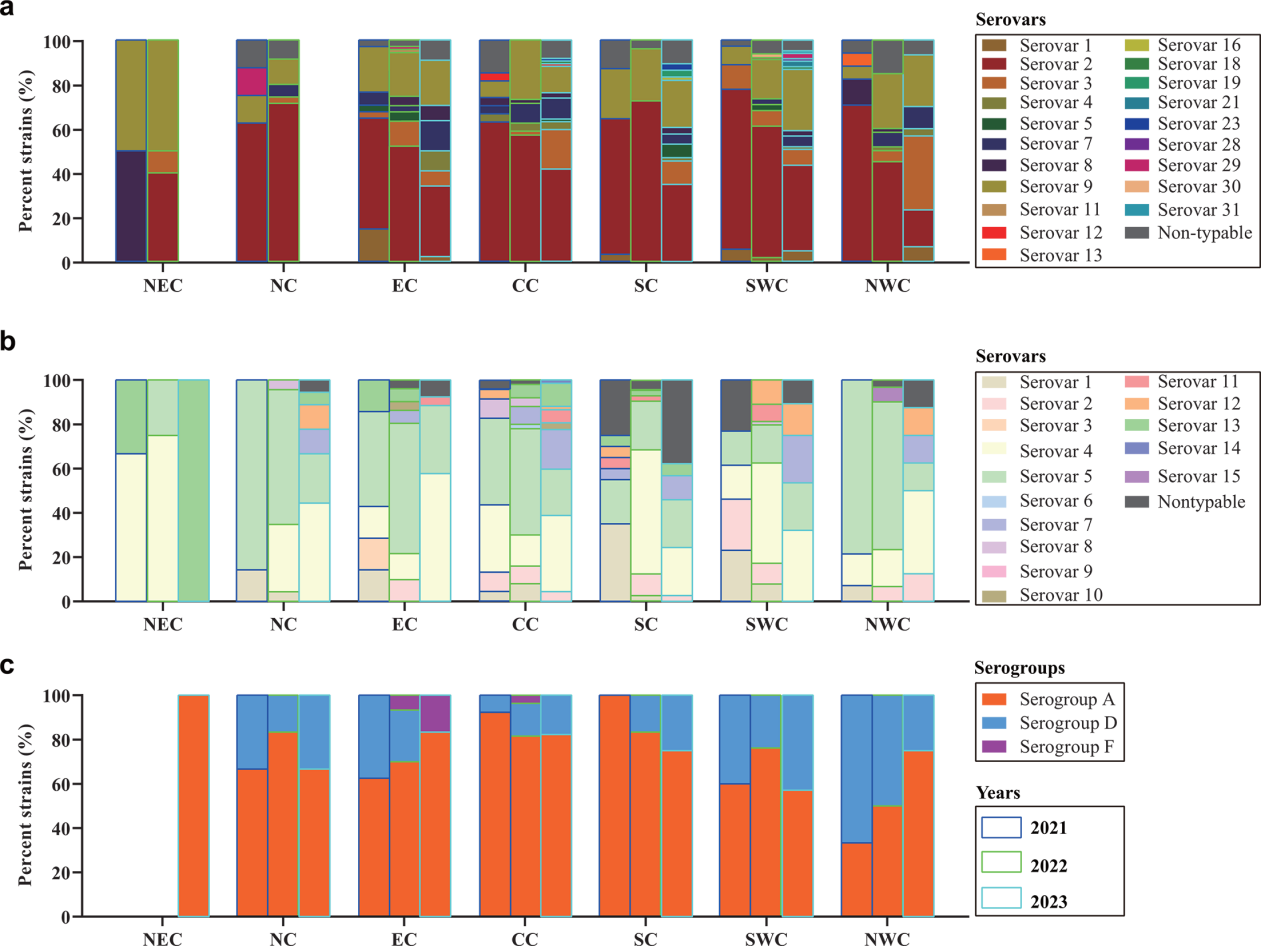
Distribution of different main bacterial serotypes associated with swine respiratory disease in China between 2021 and 2023. (**a**) Distribution of different *Streptococcus suis* serotypes associated with swine respiratory disease in different regions in China between 2021 and 2023. (**b**) Distribution of different *Glaesserella parasuis* serotypes associated with swine respiratory disease in different regions in China between 2021 and 2023. (**c**) Distribution of different *Pasteurella multocida* serotypes associated with swine respiratory disease in different regions in China between 2021 and 2023. NEC, northeast China; EC, eastern China; NC, northern China; CC, central China; SC, southern China; SWC, southwest China; NWC, northwest China.

To understand the serotypes of main respiratory bacterial pathogens prevalent on pig farms in China, we conducted a retrospective analysis of these bacterial strains isolated between 2021 and 2023. The results demonstrated that *S. suis* serovar 2 was predominantly prevalent in different Chinese regions (Fig. 6a). However, serovar 9 was also prevalent particularly in northeast China (Fig. 6a). *G. parasuis* serovars 4 and 5 were predominantly prevalent in different Chinese regions (Fig. 6b). However, serovar 14 was emerging and prevalent in regions, excluding northeast China in 2023 (Fig. 6b). *P. multocida* serogroups A and D were predominantly prevalent in different Chinese regions, and serogroup A was more predominant than serogroup D (Fig. 6c). Notably, serogroup F was also prevalent on pig farms in eastern and central China (Fig. 6c).

### Identification of antibiotic resistome

**Fig 7:**
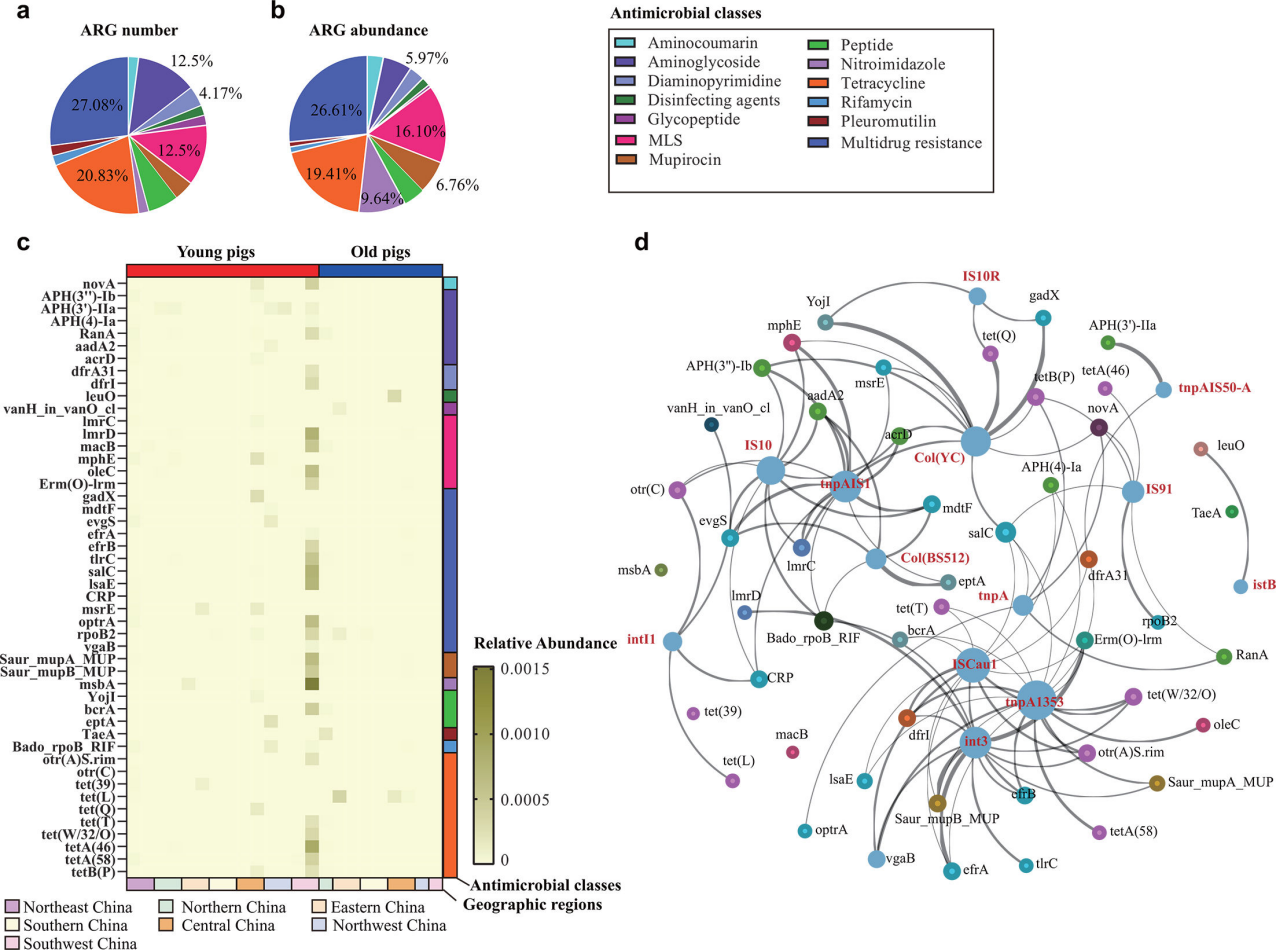
Distribution of antimicrobial resistance genes (ARGs) and their associations with mobile genetic elements (MGEs) in the lungs of pigs with respiratory disease in China. (**a**) A pie chart showing the distribution of ARGs conferring resistance to different antimicrobial classes. (**b**) A pie chart showing the abundances of ARGs conferring resistance to different antimicrobial classes. (**c**) A heatmap showing the abundances of different ARGs in the lungs of pigs with respiratory disease in different Chinese regions. (**d**) A net-chart showing the associations with ARGs and MGEs identified in the lungs of pigs with respiratory disease in China. The net-chart was generated based on Pearson’s correlation analysis. The size of each node was proportional to the number of connections, and the thickness of each edge was proportional to the Pearson’s rho value. ARGs are labeled in black, while MGEs are labeled in red.

Using the metagenome data of lung microbiome from 111 pigs that died from respiratory disease across China, we identified 48 antibiotic resistance protein-coding genes by aligning against the Comprehensive Antibiotic Resistance Database (CARD). These 48 ARGs conferred resistance to 13 predicted antimicrobial classes (Fig. 7a through c; Table S8). Among them, tetracycline resistance genes exhibited a higher frequency and abundance of examination compared with ARGs associated with resistance against the other classes, followed by macrolide–lincosamide–streptogramin B (MLS) resistance genes and aminoglycoside resistance genes (Fig. 7a and b). In addition, a proportion of genes (*efrA*, *efrB*, *evgS*, *gadX*, and *mdtF*) were found to confer resistance to multi-antimicrobial classes, including fluoroquinolones, macrolides, and rifamycin or penams, simultaneously (Fig. 7a through c). Notably, genes, such as *leuO*, which are responsible for mediating resistance to disinfecting agents were also examined (Fig. 7a through c). Our further analysis demonstrated that many of these ARGs were significantly associated with a substantial number of mobile genetic elements (MGEs), including transposons (e.g., *tnp*AIS1, *tnp*A1353, *int*3, and *IS*Cau1) and plasmids [e.g., Col(BS512), Col(YC)], identified in the pulmonary metagenomes determined in this study (Fig. 7d).

## DISCUSSION

To the global pig industry, respiratory disease not only threatens pig health but also leads to production and economic losses due to reduced growth rates and a lower feed conversion efficiency (4). While cases of respiratory disease induced by non-infectious factors can be observed, the majority of cases are caused by pathogenic microbes, particularly viruses and bacteria, with coinfections being common (16, 18, 19). However, limited knowledge has been gained regarding the “whole pathogens” and their “coinfection patterns” in swine respiratory disease at a large-scale farm level. This might be partially due to the complexity of on-site investigations at farms and unavailability of high-throughput strategies capturing all pathogens in one sweep in veterinary clinic practice. Recently, particularly after the COVID-19 outbreak, the combined use of metatranscriptomic sequencing and metagenomic sequencing has been successful in discovering multiple pathogenic microbes simultaneously from a multitude of samples (13, 14), enabling the identification of the “whole pathogens” responsible for swine respiratory disease and revealing interactions between different agents on farm scale.

By conducting metatranscriptomic sequencing combined with metagenomic sequencing, it is not surprising that PRRSV was identified as a primary agent for swine respiratory disease, with high abundance and broad geographic regions of detection. Indeed, PRRSV has continued to be one of the most harmful pathogens on pig farms in China and has caused massive economic losses to the Chinese pig industry since the first report of PRRSV strain CH-1a in China in 1996 (20). PRRSV strains are divided into PRRSV1 and PRRSV2 (21). While PRRSV1 strains have been identified on pig farms in China (22, 23), PRRSV2 has been the predominantly prevalent type (24, 25). Consistently, PRRSV strains characterized by metatranscriptomic sequencing or retrospective analysis in this study were identified as PRRSV2, whereas a small number of PRRSV strains retrospectively analyzed between 2018 and 2023 belonged to PRRSV1. It has also been reported that currently four major lineages (L1, L3, L5, and L8) of PRRSV2 strains are prevalent in China, with L1 becoming dominant over L8 after 2017 (26). In agreement with these findings, our study also characterized L1 as the dominant lineage. In addition to PRRSV, PCV2 has also been recognized as a primary viral agent for swine respiratory disease (16). Consistently, PCV2 was identified in lung tissues by metatranscriptomic sequencing, indicating this type of PCV remains an important respiratory virus on pig farms in China. However, our retrospective analysis demonstrated that other types of PCV, including PCV1, PCV3, and PCV4, were also prevalent on Chinese pig farms. Previous studies have shown that PCV1 is nonpathogenic in pigs, but the newly emerged PCV3 and PCV4 could cause various symptoms in pigs in addition to respiratory lesions (27, 28). The harms of PCV3 and PCV4 are increasing in the global pig industry (29, 30), and continuous monitoring the prevalence of these two types of PCVs is necessary.

Our analysis indicated the prevalence of heterogeneous groups of IAV, particularly H1N1 and H1N2, which were responsible for swine respiratory disease. Although several studies have pointed out that swine influenza is one of the most important diseases in global pig industry (31), the prevalence of IAV in pigs has not received much attention, and worldwide structured surveillance on swine influenzas is weak (32). This is partially because swine influenzas are not considered as a major threat to animal health, according to the European Centre for Disease Prevention and Control (32). However, the potential harmful impact of IAV on pig industry should not be ignored, as this viral species can cause secondary bacterial infection, thereby increasing the morbidity and mortality (33). It should be noted that heterogeneous groups of PPVs (PPV1~PPV8) were also identified in this study. Among these, PPV2 has been reported to be predominantly associated with macrophages in PRDC (34), and PPV7 has been reported to have coinfections with PCV2, PCV3, and PRRSV (20). Further exploration is needed to understand the role of other PPV types in swine respiratory disease. In addition to above mentioned viruses, the identification of GETV and PRCoV should be noted. GETV is a mosquito-borne zoonotic arbovirus and has been described to cause diarrhea and death in piglets, reproductive failure, and abortion in sows (35, 36). While the prevalence of GETV on farms in China has been documented (35), recent knowledge about the role of this virus in swine respiratory disease is still limited. PRCoV characterization from pigs has been reported in Korea and the United States (37, 38), but limited information has been obtained on Chinese pig farms. Further monitoring and investigations are necessary to understand their impact on pig health.

A substantial number of bacteria were also identified in this study. It is not surprising to identify *S. sui*, *G. parasuis*, and *P. multocida* as they rank among the top three isolated bacterial species from Chinese pig farms every year, and they are all associated with swine respiratory disease (39). Notably, our retrospective serotyping assays of these three species demonstrated a complex situation regarding the prevalent bacterial serotypes on Chinese pig farms, and the identification of several unusual serotypes should receive attention. For example, *P. multocida* serotype F is not frequently characterized on pig farms, but they may display high virulence to pigs (40). The emergence of these novel strains may increase the difficulty of preventing and controlling bacterial infections. It is also not surprising to identify *M. hyopneumoniae* from the lungs of pigs with respiratory disease since the prevalence of this species on Chinese pig farms is also common in recent years (41). While *M. hyorhinis* was also identified, the prevalent data of this species on Chinese pig farms remain limited, and further investigations are necessary. Another two notable bacterial species identified in this study were *E. coli* and *K. pneumoniae*. Recent studies have revealed a high frequency of recovering these two species of *Enterobacteriaceae* from pig farms, even from pig lungs (42, 43). However, laboratory studies are still necessary to determine whether the challenge of them could induce swine respiratory disease. It is worth noting that both of these two species of *Enterobacteriaceae* are frequently associated with human pulmonary infections (44, 45). Additionally, the prevalence of *E. coli* and *K. pneumoniae* on pig farms may also accelerate the spread of antimicrobial resistance, as these two *Enterobacteriaceae* species are recognized as important shuttles for mobile ARGs (46, 47).

Our analysis also demonstrated a complex situation of pathogen–pathogen interactions in swine respiratory disease. It has been documented that swine respiratory disease usually involves coinfections of multiple pathogens (10). While the mechanisms of interactions between several of these pathogens have been elucidated recently (48, 49), further investigations are necessary. Respiratory disease has also been recognized as a main reason for antimicrobial usage in pig industry (4, 50, 51). Antimicrobials, such as tetracyclines, aminoglycosides, and macrolides, are frequently used against swine respiratory disease (52). Correspondingly, the abundances of ARGs conferring resistance against these classes are relatively high, as determined in this study. Notably, we also found that these ARGs displayed a correlation with multiple MGEs identified in the lungs of pigs with respiratory disease. These results agree well with those from a recent study (15), which might increase the risk of the spread of these ARGs.

Although this work is limited by our inability to include more lung tissues from more farms in a wider range of Chinese provinces due to the strict biosecurity requirements for combating the prevalence of African swine fever, and the COVID-19 pandemic between 2019 and 2020 also limited our ability to collect more bacterial strains for retrospective investigation, our work still provides comprehensive knowledge about the viral and bacterial pathogens associated with swine respiratory disease on farms in China at a nationwide scale. Another limitation of our study might be the use of sample pooling for sequencing due to the high cost of sequencing individual samples. This strategy may have an impact on the accuracy of quantification of pathogens or ARGs. However, pooling samples is still an acceptable strategy due to its low cost and applied by many studies (53–55).

In conclusion, we reported the identification of viruses and bacteria associated with swine respiratory disease by integrating metatranscriptomic sequencing and metagenomic sequencing. Overall, PRRSV, PCV, IAV, *S. suis*, *G. parasuis*, *P. multocida*, *M. hyopneumoniae*, and *M. hyorhinis* were identified as the main pathogens for swine respiratory disease. However, several other microbes, such as GETV, PRCoV, *E. coli*, and *K. pneumoniae* may also contribute to the disease. In addition, a complex interaction between different microbes involving in swine respiratory disease was demonstrated. Our work may be beneficial for further understanding the etiology, epidemiology, and microbial interactions in swine respiratory disease, and may also shed a light on the development of effective vaccines against the disease.

## MATERIALS AND METHODS

### Sample collection and preparation of sequencing pools

Between 1 October 2022 and 31 October 2023, we collected 111 lungs of pigs, comprising 70 lungs from pigs aged younger than 70 days (designed young pigs) and 41 lungs from pigs aged older than 70 days (designed old pigs) from farms in 25 provinces in different regions in China (northeast China: Heilongjiang, Liaoning; northern China: Hebei, Inner Mongolia, Shanxi, Tianjin; eastern China: Anhui, Fujian, Jiangsu, Jiangxi, Shandong, Zhejiang; central China: Henan, Hubei, Hunan; southern China: Guangdong, Guangxi; southwest China: Chongqing, Guizhou, Sichuan, Yunnan; and northwest China: Gansu, Ningxia, Shaanxi, Xinjiang) (Fig. S1a). We divided the pigs into two age groups based on the 70-day threshold, as pigs typically enter the fattening stage around this age, a period during which significant changes in immune programs and growth environments occur (56). Subsequently, these samples were grouped into 23 sequencing pools according to the ages of the pigs as well as the geographical regions (northeast China, northern China, eastern China, central China, southern China, southwest China, and northwest China) where the samples were collected (Fig. S1b).

### Library preparation and Illumina sequencing

Genomic DNAs were extracted from different sample pools using a TIANamp stool DNA kit (Tiangen, Beijing, China) following the manufactory’s instructions, while total RNAs were extracted using a QIAamp MinElute Virus Spin Kit (Qiagen, Hilden, Germany) following the manufactory’s instructions. Subsequently, a VAHTS Universal Plus DNA Library Prep Kit for Illumina (Vazyme, Nanjing, China) was used to prepare sequencing libraries for metagenomic sequencing using the extracted genomic DNAs. The libraries were then assessed using an Agilent 4200 Bioanalyzer (Agilent, Santa Clara, CA, USA) and Qubit Fluorometric Quantification (ThermoFisher, Waltham, MA, USA). Qualified libraries were finally sequenced on an Illumina Nova Seq 6000 platform (Illumina, San Diego, CA, USA) using the 2 × 150 bp paired-end protocol.

To prepare libraries for metatranscriptomic sequencing, the sequence independent amplification (SIA) protocol described previously was followed (57). Briefly, the extracted RNAs were quantified using a Equalbit RNA HS Assay Kit (Vazyme, Nanjing, China) on Qubit. The RNA samples were reverse transcribed using 200 U of Superscript III reverse transcriptase (Life Technologies, Carlsbad, CA, USA) with a primK random (5′-GACCATCTAGCGACCTCCACNNNNNNNN-3′). Subsequently, the second strand of cDNA synthesis was performed using Klenow enzyme (NEB, Ipswich, MA, USA). The synthesized second-strand cDNA was used as a template for PCR amplification with specific primers (5′-GACCATCTAGCGACCTCCAC-3′). Following PCR amplification, the products were purified using magnetic beads. The purified PCR products were used for DNA library construction, which involved enzyme digestion, end repair, A-tailing, adapter ligation, and PCR amplification. The libraries were constructed using the VAHTS Universal Plus DNA Library Prep Kit for Illumina (Vazyme, Nanjing, China). The quality of the library was assessed using the Agilent 4200 Bioanalyzer (Agilent, Santa Clara, CA, USA), and quantification was performed using the Qubit fluorometer (ThermoFisher, Waltham, MA, USA). Qualified libraries were subjected to 2 × 150 bp paired-end sequencing on the Illumina Nova Seq 6000 platform (Illumina, San Diego, CA, USA). Both metatranscriptomic and metagenomic sequencing were conducted at Chengdu Life Baseline Technology Co., LTD.

### Bioinformatic analysis

After sequencing, the raw data were processed using BBDuk software to eliminate low-quality reads and adapters. The clean reads were evaluated using FastQC and then aligned to pig reference genomes with Bowtie 2 (58). After filtering out host genomes, the data (effective reads) underwent *de novo* assembly with metaSPAdes (59) to produce contigs. Open reading frames (ORFs) contained within different contigs were identified using Prodigal (60). The generated contigs and predicted ORFs were aligned to the NCBI non-redundant (NR) database using Diamond (e-value: 1e−5) (61) to identify viral and bacterial sequences. Candidate viral sequences were further aligned to the NCBI nucleotide database via blastn, with a sequence not being assigned as a viral sequence if the blastn results indicated that the non-viral portion of this sequence exceeded 20%. For sequences that could not be confirmed by blastn, the candidate viral sequences were analyzed using VirBot (62). ARGs were identified by aligning the predicted ORFs against the CARD database (e-value: 1e−5) (63). MGEs were identified using the MobileGeneticElement Database (64). Phylogenetic trees were generated using MEGA11 (65) through the neighbor-joining algorithm with 1,000 bootstrapping.

### Molecular docking analysis

Molecular docking analysis was performed to investigate the interaction between the spike protein of PRCoV strain HDD2 and the porcine respiratory receptor ACE2, as well as the porcine intestinal receptor APN. Following established protocols, the three-dimensional structures of the spike protein, ACE2, and APN were predicted using AlphaFold3 (66). Molecular docking simulations were then conducted, with the spike protein as the ligand and ACE2 and APN as the receptors. Docking results were visualized using PyMOL 3.03 (67). The predicted oligomeric structures were further assessed using PRODIGY (https://nestor.science.uu.nl/prodigy/) to calculate the binding energies of the protein complexes.

### PCR assays for bacterial serotyping

Serotypes of *S. sui*, *G. parasuis*, and *P. multocida* were determined using multiplex PCR methods following the protocols with the primers described by Liu et al. (68), Howell et al. (69), and Townsend et al. (70), respectively.

### Statistical analysis

The relative abundance level for each bacterial/viral genome was calculated using the following formula: total bacterial/viral reads/total reads in the sample × 1,000,000, which corresponds to reads per million (RPM). The Mann−Whitney *U* test was employed to evaluate continuous variables that did not follow a normal distribution (53, 71). This analysis was performed using the “scipy.stats” package in Python. *P*-values for multiple comparisons were corrected using the Benjamini−Hochberg method.

## Data Availability

Raw data generated from metatranscriptomic and metagenomic sequencing are deposited into NCBI SRA database. The project number is PRJNA1131162. Accession numbers are SRR29720838, SRR29720839, SRR29720840, SRR29720841, SRR29720842, SRR29720843, SRR29720844, SRR29720845, SRR29720846, SRR29720847, SRR29720848, SRR29720849, SRR29720850, SRR29720851, SRR29720852, SRR29720853, SRR29720854, SRR29720855, SRR29720856, SRR29720857, SRR29720858, SRR29720859, SRR29720860. Accession numbers for the complete genome sequences of Getah virus (strain XNX2) and Zhejiang porcine bastro-like virus (strain HDD2) identified in this study were PQ106803 and PQ106802, respectively.

## SUPPLEMENTAL MATERIAL

Fig. S1 msystems.00025-25-s0001.pdf Sample collection and data generated from Illumina sequencing.

Fig. S2 msystems.00025-25-s0002.pdf Phylogenetic relationships of the Zhejiang porcine bastro-like virus (strain HDD2) identified in this study and the other viruses in the family Astroviridae.

Table S1 msystems.00025-25-s0003.xls Relative abundances of viral species associated with swine respiratory disease in China.

Table S2 msystems.00025-25-s0004.xls Relative abundances of bacterial species associated with swine respiratory disease in China.

Table S3 msystems.00025-25-s0005.xls PRRSV strains used for the retrospective analysis in this study.

Table S4 msystems.00025-25-s0006.xls IAV strains used for the retrospective analysis in this study.

Table S5 msystems.00025-25-s0007.xls PCV strains used for the retrospective analysis in this study.

Table S6 msystems.00025-25-s0008.xls PPV strains used for the retrospective analysis in this study.

Table S7 msystems.00025-25-s0009.xls GETV strains used for retrospective analysis in this study.

Table S8 msystems.00025-25-s0010.xls Antimicrobial resistance genes identified in this study.

ASM does not own the copyrights to Supplemental Material that may be linked to, or accessed through, an article. The authors have granted ASM a non-exclusive, world-wide license to publish the Supplemental Material files. Please contact the corresponding author directly for reuse.
