# Supplementary material for: Discovery of viruses and bacteria associated with swine respiratory disease on farms at a nationwide scale in China using metatranscriptomic and metagenomic sequencing
Source: mSystems. 2025 Jan 30;10(2):e00025-25. doi: 10.1128/msystems.00025-25 (PMC11834406; doi:10.1128/msystems.00025-25)
Supplement: Fig. S2 — Phylogenetic relationships of the Zhejiang porcine bastro-like virus (strain HDD2) identified in this study and the other viruses in the family Astroviridae. [file msystems.00025-25-s0002.pdf]

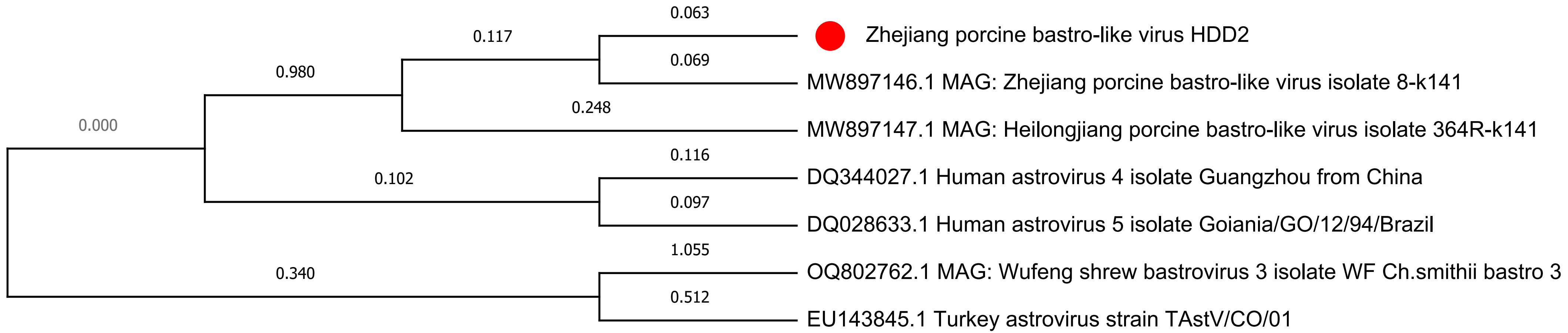

Figure S2. Phylogenetic relationships of the Zhejiang porcine bastro-like virus (strain HDD2) identified in this study and the other viruses in the family *Astroviridae*.
