# Supplementary material for: Discovery of viruses and bacteria associated with swine respiratory disease on farms at a nationwide scale in China using metatranscriptomic and metagenomic sequencing
Source: mSystems. 2025 Jan 30;10(2):e00025-25. doi: 10.1128/msystems.00025-25 (PMC11834406; doi:10.1128/msystems.00025-25)
Supplement: Fig. S1 — Sample collection and data generated from Illumina sequencing. [file msystems.00025-25-s0001.pdf]

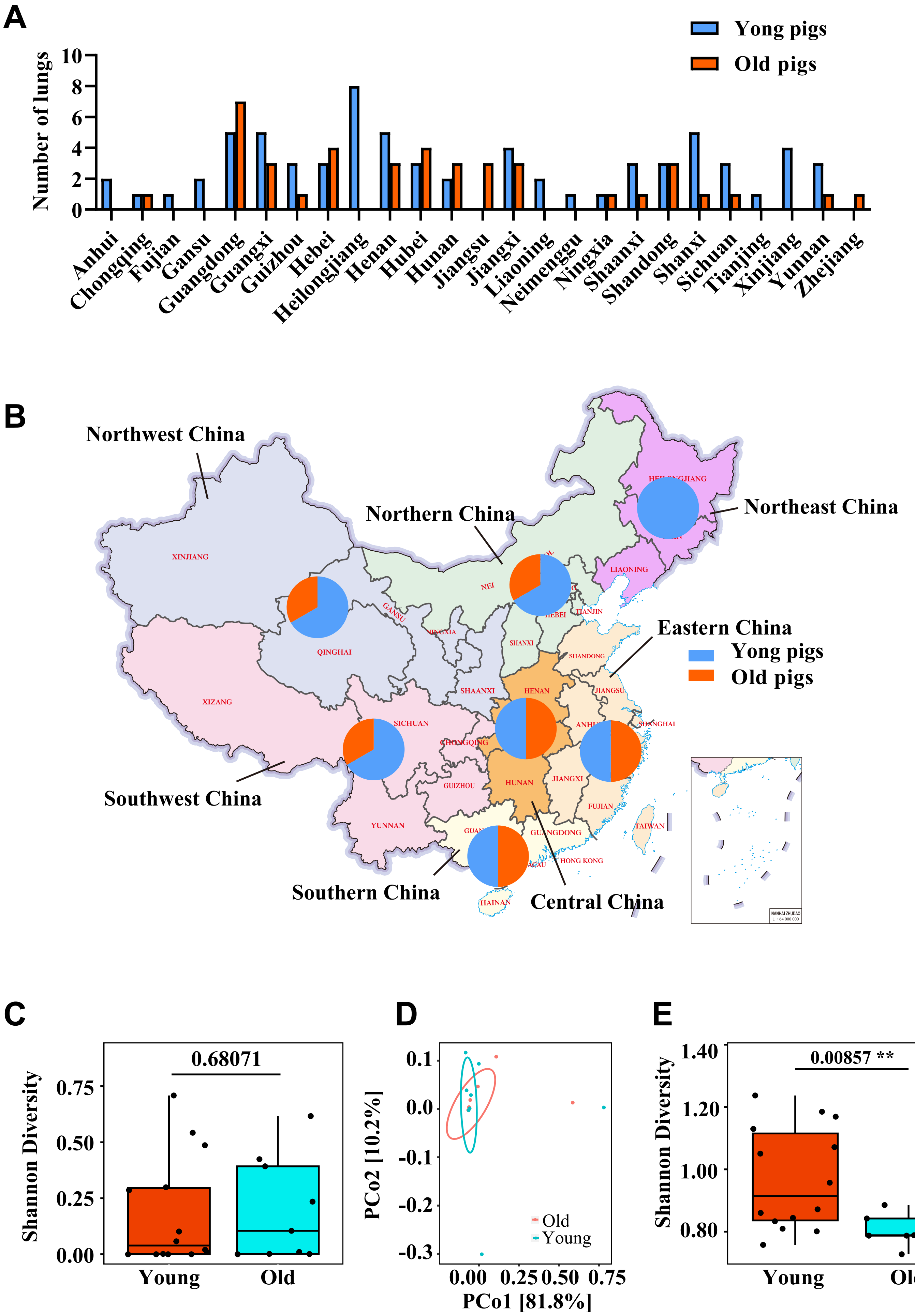

Figure S1. Sample collection and data generated from Illumina sequencing. Panels **A** and **B** show the number of samples collected from pigs with respiratory diseases in different regions in China. Panels **C** and **D** display the Shannon diversity and/or principal-component (PC) diversity of metatranscriptomic sequencing data generated from the lungs of young pigs and old pigs in China. Panels **E** and **F** display the Shannon diversity and/or principal-component (PC) diversity of metagenomic sequencing data generated from the lungs of young pigs and old pigs in China.
